# Physiological Response of *Saccharomyces cerevisiae* to Silver Stress

**DOI:** 10.3390/jof8050539

**Published:** 2022-05-22

**Authors:** Janelle R. Robinson, Omoanghe S. Isikhuemhen, Felicia N. Anike, Kiran Subedi

**Affiliations:** 1 Department of Natural Resources and Environmental Design, North Carolina Agricultural and Technical State University, 1601 East Market Street, Greensboro, NC 27411, USA; jrrobin3@aggies.ncat.edu (J.R.R.); fnanike@ncat.edu (F.N.A.); 2 Analytical Services Laboratory, College of Agriculture and Environmental Sciences, North Carolina Agricultural and Technical State University, 1601 East Market Street, Greensboro, NC 27411, USA; ksubedi@ncat.edu

**Keywords:** *Saccharomyces cerevisiae*, silver nitrate, silver nanoparticle, physiological response, mycosynthesis

## Abstract

Silver nanoparticle (AgNP) production and their use as antimicrobial agents is a current area of active research. Biosynthesis is the most sustainable production method, and fungi have become candidates of interest in AgNP production. However, investigations into the physiological responses of fungi due to silver exposure are scanty. This present work utilized two strains of *Saccharomyces cerevisiae* (one used in commercial fermentation and a naturally occurring strain) to determine the physiological consequences of their transient exposure to AgNO_3_. The assessments were based on studies involving growth curves, minimal inhibitory concentration assays, scanning electron microscopy (SEM) and transmission electron microscopy (TEM) imaging, and inductively coupled plasma optical emission spectroscopy (ICP-OES). Results indicated (a) the capability of *S. cerevisiae* to produce silver nanoparticles, even at elevated levels of exposure; (b) strain origin had no significant impact on *S. cerevisiae* physiological response to AgNO_3_; and (c) coexposure to copper and silver significantly increased intracellular copper, silver, and calcium in treated yeast cells. In addition, electron microscopy and ICP-OES results revealed that both strains internalized silver after exposure, resulting in the shrunken and distorted physical appearance visible on SEM micrographs of treated cells. Though a promising candidate for AgNPs biosynthesis, this study analyzed the effects of transient silver exposure on *S. cerevisiae* growth physiology and morphology.

## 1. Introduction

The incidence of fungal–silver interactions is greatly increasing due to the growing interest in the use of fungi for silver nanoparticle (AgNP) production [1,2,3]. It is predicted that AgNPs will have an increased compound annual growth rate of 15.7% between 2020 and 2027 [4]. With some fungi having a high metal-tolerance capacity and the active research on AgNP mycosynthesis, it is logical to expect that fungi will play a significant role in the predicted market increase [5,6]. Species such as *Fusarium oxysporum*, *Botryodiplodia theobromae*, *Penicillium oxalicum*, and Saccharomyces cerevisiae have been investigated for their ability to produce AgNPs, the resulting AgNP characteristics, and levels of toxicity [7,8,9,10]. In addition, mycosynthesized AgNPs have been studied for their use as antimicrobial agents against medically relevant microorganisms, as synergistic enhancers for antibiotics, and as a promising treatment option for cancer therapy [11,12,13,14,15]. These types of fungal–silver interactions benefit humans in multiple ways. However, there is a lack of knowledge about how metal ions affect the morphology and physiology of different fungi, even in the common yeast *S. cerevisiae,* which has vast industrial applications and economic importance. Thus far, research has shown some impacts of silver exposure, including effects on homeostatic systems and some physiological responses [16,17,18,19]. Transcriptome analysis has demonstrated that exposure can downregulate genes involved in the synthesis of membrane components, and imaging studies have shown that exposure can alter cellular morphology, even reducing dimorphism in pathogenic *Candida* sp. [16,20,21,22,23]. Most studies also utilize well-annotated laboratory strains of *S. cerevisiae* and pathogenic *Candida* spp.; however, reports on the impacts on most other fungal strains are limited.

Additionally, there is little understanding about silver exposure and silver uptake, capacity in *S. cerevisiae*. A recent review on this topic revealed that while some mechanisms of homeostasis have been unveiled, there is no consensus on the exact mechanisms [24]. The present study used two different strains of *S. cerevisiae*, one implemented in commercial fermentation processes and the other naturally present in palm wine from Ebelle, Nigeria. They were used to explore the physiological impacts of transient silver exposure and if the origin of each strain impacted the response. With the knowledge that both AgNPs and AgNO_3_ can induce toxicity via the release of silver ions, AgNO_3_ was used in the experiments.

## 2. Materials and Methods

### 2.1. Saccharomyces cerevisiae Strains and Growth Conditions

Two *S. cerevisiae* strains were obtained for this work. The first strain, termed commercial strain, was SUPERSTART^TM^ active dry yeast obtained from Lallemand Biofules and Distilled Spirits, a yeast designed for commercial production of biofuels or spirits. The second strain, termed naïve strain, was isolated from Ebelle palm wine, a naturally fermented alcoholic beverage produced from the collection of sap from the African oil palm, *Elaeis guineensis* Jacq. in Ebelle, Nigeria. The palm wine is made by using a container made from *Lagenaria siceraria* (calabash fruit) to collect the sap drippings from the holes made at the base of the inflorescence of the oil palm (Arecaceae family). Yeast existing in the calabash from previous use and yeast from the environment ferment the juice from the oil palm inside the calabash. Therefore, the yeast from Ebelle palm wine had no prior exposure to metal compared to those used for commercial fermentation processes. For identification, yeast underwent Sanger sequencing via Eurofins Genomics with primers ITS1F (CTTGGTCATTTAGAGGAAGTAA) and ITS4 (TCCTCCGCTTATTGATATGC). The National Center for Biotechnology Information (NCBI) Basic Local Alignment Search Tool (BLAST) returned the identification of commercial and naïve yeast as *S. cerevisiae* and with accession numbers CP006391.1 and LC215451.1, respectively [25]. In all experiments, yeasts in liquid media (20 g/L dextrose, 5 g/L yeast extract) were grown at 30 °C, 150 RPM in 50 mL Erlenmeyer flasks (Pyrex, Corning, Corning, NY, USA) in a shaking incubator (Thermo Scientific MAXQ 4000, Waltham, MA, USA). The cultures on solid media (20 g/L dextrose, 5 g/L yeast extract, and 15 g/L agar) were grown in a stationary incubator (Fisher Scientific 146E, Waltham, MA, USA) at 30 °C in 100 mm × 15 mm Petri dishes. 

### 2.2. Chemicals and Reagents

Silver nitrate (Alfa Aesar ACS 99.9% +) was made into 1000 mg/L stock solution of AgNO_3_ and was used for all experiments that required AgNO_3_. All yeast were cultured on media that contained 20 g/L dextrose, 5 g/L yeast extract, and 15 g/L agar (solid media). Silver, calcium, and copper standards (1000 ppm each) purchased from High-Purity standards (Charleston, SC, USA) were used for elemental analysis.

### 2.3. Minimal Inhibitory Concentration Assay 

The minimal inhibitory concentration (MIC) is the lowest concentration of AgNO_3,_ which completely inhibits cell growth. Before the MIC assay, cells were grown overnight in liquid media (20 g/L dextrose, 5 g/L yeast extract). After overnight growth, commercial yeast (*n* = 10) and naïve yeast (*n* = 10) were used to inoculate 96-well plates, with the final yeast OD_A600_ at 0.1 and incubated with AgNO_3_ at concentrations of 0, 10, 20, 22.5, 25, 27.5, and 30 mg/L. 0 mg/L AgNO_3_ (no addition of AgNO_3_) was the positive control. Negative controls were all reported concentrations with no yeast cells. Plates were incubated at 30 °C and 150 RPM for 48 h. After incubation, cells were subjected to 10-fold serial dilutions, spread on solid media, and incubated at 30 °C for 48 h to obtain single colonies. The lowest AgNO_3_ concentration that resulted in no visible colony formation was selected as the MIC. The next lowest concentration was selected as the sub-MIC. The sub-MIC was toxic enough to inhibit a significant amount of growth without completely preventing growth. AgNO_3_ concentration at sub-MIC was used for subsequent experiments.

### 2.4. Phenotypic Screening: Selection of Naturally Sensitive Isolates

**Figure 1:**
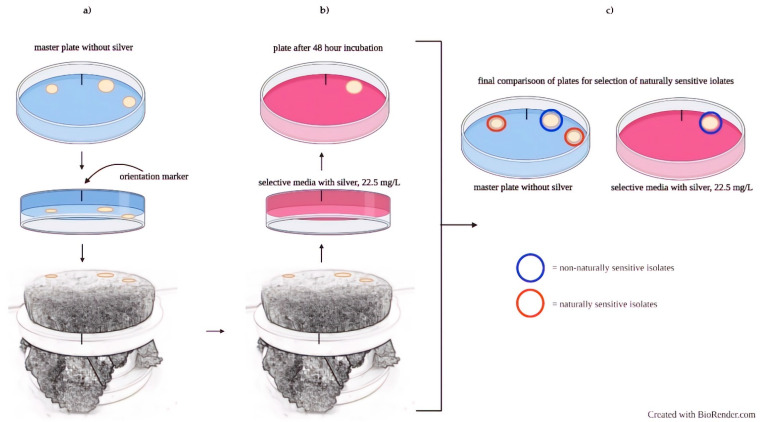
Replica plating for the selection of naturally sensitive isolates. (**a**) Single colonies from nonselective media impressed onto velvet. (**b**) Selective media containing silver received the impression from the velvet. (**c**) Incubation of selective media determined NS isolates on nonselective media.

The sub-MIC was used to select naturally sensitive (NS) isolates from the commercial and naïve yeast (i.e., isolates unable to grow at the sub-MIC) via replica plating [26,27]. Commercial and naïve yeast were grown overnight in liquid media, serially diluted, and spread on solid, nonselective media (master plate) for single colonies. The master plate was replica-plated onto an apparatus that held sterile velvet squares (Figure 1). The velvet then had an impression of the master plate. The plate with silver-containing selective media was pressed on top of the velvet with the master-plate impression. The impression was then imprinted onto the selective media and incubated for 48 h at 30 °C. After 48 h, the isolates that grew on the master plate but not the selective media were selected as naturally sensitive isolates. Ten NS commercial isolates (NS C1–C10) and ten NS naïve isolates (NS N1–N10) were selected for further experiments. This selection ensured the isolates chosen did not contain any preexisting natural resistance to the sub-MIC. All 20 isolates were grown in liquid culture and stored at a 1:1 ratio in 30% glycerol (Arcos Organics 99.9 + %) at −80 °C.

### 2.5. Growth Curve Assay

Growth curve assays were conducted to determine the growth pattern of NS isolates with and without silver. In a 96-well plate (Costar 96-well Flat Bottom Assay Plate), 10 NS commercial yeast and 10 NS naïve yeast isolates were exposed to the sub-MIC and media without AgNO_3_ and incubated inside a BioTek Synergy HY Plate Reader. The plate reader was set at 30 °C and used absorbance at 600 nm to collect a data point every hour for 72 h.

### 2.6. Scanning Eletron Microscopy (SEM) and Transmission Eletron Microscopy (TEM)

NS commercial yeast isolates (C1–C5) and NS naïve yeast isolates (N1–N5) were grown in 40 mL liquid media at the sub-MIC and in media without AgNO_3_ for 48 h (Table 1). Samples were then delivered to the North Carolina State University Analytical Instrumentation Facility for SEM and TEM preparation and imaging. Sample preparation was based on several existing protocols [28,29,30,31]. All samples were pelleted and resuspended in 3% glutaraldehyde in a 0.1 M sodium cacodylate buffer. To prepare samples for SEM imaging, 100 µL of the suspended cells were mixed into 1 mL of 0.1 M sodium cacodylate buffer and filtered through a 0.4 µm nucleopore filter. The filtrate was discarded, and the filter was soaked in a container, on ice, with the same buffer for 15 min. The filter was moved twice to two new containers with the same buffer, each for another 15 min of incubation time. The filter was then dehydrated by washing samples with 30% and 50% ethanol (1 h each) and then placed into 70% ethanol at 4 °C for 2 h. The filter was then washed in cold 95% ethanol and cold 100% ethanol for 1 h each and washed twice at room temperature, in 100% ethanol. Samples on the filter then underwent critical-point drying in liquid CO_2_. Each filter was then split and mounted on separate stubs and was left uncoated. For TEM imaging, samples in the 0.1 M sodium cacodylate buffer were pelleted, and the supernatant was removed. The samples were washed three times (15 min each) in the same buffer, pelleted, and the supernatant removed. Samples were then fixed in 0.1 M sodium cacodylate buffer with 2% osmium tetroxide for 4 h at 4 °C. Samples were then washed three more times as described above, pelleted, and had a 2% agarose solution added. Agarose was removed after 30 min, and samples underwent six 24 h washes in ethanol (50%, 70%, 95%, 100%, 100%, and 100%). Samples were then embedded in 50% Spurrs (epoxide resin with low viscosity) and washed in 100% ethanol for 24 h. This was repeated with 75% Spurrs, before samples were embedded in 100% Spurrs in BEEM capsules and incubated 70 °C overnight. Capsules were removed and samples sectioned with a Leica UC7 Cryo Ultramicrotome at 100 nm thickness. SEM imaging was completed on the FEI Verios 460 L field-emission scanning electron microscope (FESEM). TEM imaging was conducted on a Talos F200X G2.

### 2.7. Inductively Coupled Plasma Optical Emission Spectroscopy (ICP-OES) 

ICP-OES was used to monitor the cellular influx of silver into yeast during silver exposure. Due to the potential use of copper transport channels by silver ions and the importance of calcium to cellular functioning, ICP-OES was also used to analyze the influx of copper (II) sulfate and calcium chloride [24]. NS commercial yeast isolates, C1–C5, and NS naïve yeast isolates, N1–N5, were grown overnight in liquid media and used to inoculate flasks that contained 22.5 mg/L AgNO_3_, 22.5 mg/L CuSO_4_, 22.5 mg/L CaCl_2_, or 22.5 mg/L of both AgNO_3_ and CuSO_4_, with the final yeast OD_A600_ at 0.1. 

Yeasts were incubated at 30 °C, 150 RPM for 48 h. After incubation, 20 mL of each sample was pelleted and underwent digestion. Predigestion was completed by incubating samples with nitric acid (68–70%) for 10 min; full digestion used a MARS 6 (CEM Microwave Technology Ltd., Matthews, North Carolina, USA) microwave digester with the parameters in Table 1. The clear, fully digested sample was diluted to a volume of 50 mL using double-deionized (DI) water and analyzed using Optima 8300 ICP-OES (Perkin Elmer, Inc., Shelton, CT, USA). Following digestion, samples underwent elemental ICP analysis (Table 2) using the following emission lines for Ca, Cu, and Ag quantification: Ca (II) 317.933 nm, Cu (I) 324.372 nm, and Ag (I) 328.068 nm. The digestion and ICP analysis were completed in the Analytical Services Laboratory (ASL) at North Carolina Agricultural and Technical State University.

### 2.8. Statistical Analysis 

Statistical analysis of the MIC assays and the growth curve used IBM SPSS Version 26. MIC analysis used a one-way analysis of variance (ANOVA) with a post hoc Tukey test. The growth curve analysis used a one-sample *t*-test to determine statistical significance between means. Graphs were generated with Microsoft Excel 2016.

## 3. Results 

### 3.1. Minimal Inhibitory Concentration Assay

**Figure 2:**
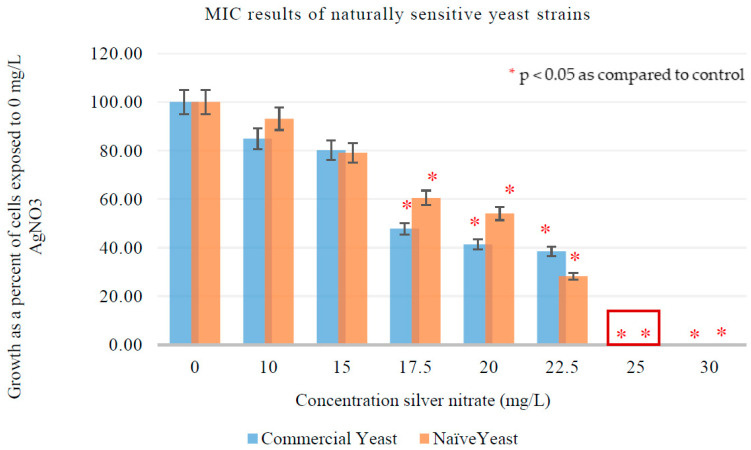
Minimal inhibitory concentration of AgNO_3_ for NS commercial (*n* = 10) and naïve (*n* = 10) yeast. The red asterisk represents AgNO_3_ concentrations that resulted in significantly less (*p* < 0.05) growth when compared to the control. The red box represents growth that occurred at the MIC. Error bars represent percentage error (5%).

MIC assays were conducted to assess the sensitivity of *S. cerevisiae* to AgNO_3_. Figure 2 represents colony growth of exposed cells as a percentage of the growth of cells that did not receive any AgNO_3_ treatment. In both strains, no significant inhibition activity occurred at 0, 10, and 15 mg/L AgNO_3_. Significant growth inhibition began at 17.5 mg/L AgNO_3_. There was no observed growth at 25 and 30 mg/L AgNO_3_, which indicated both yeast strains had a MIC of 25 mg/L AgNO_3_ and a sub-MIC of 22.5 mg/L AgNO_3_. At the sub-MIC, NS commercial and naïve yeast had comparable growth.

### 3.2. Growth Curve Assay

**Figure 3:**
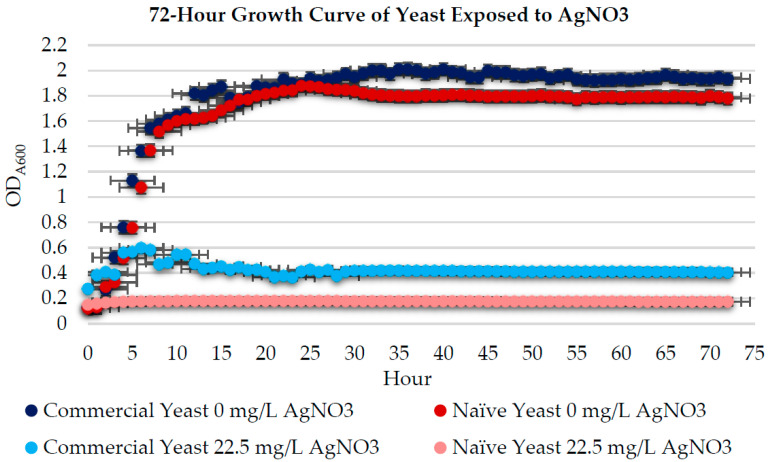
Growth curves for NS commercial (*n* = 24) and naïve (*n* = 24) yeast exposed to 0 mg/L AgNO_3_ and the sub-MIC (22.5 mg/L AgNO_3_). Error bars represent percentage error (5%).

After determining the sub-MIC, both yeast strains underwent growth curve assays to determine if silver exposure affected the normal growth pattern. Figure 3 shows the 72 h growth curve of NS commercial yeast (*n* = 10) and NS naïve yeast (*n* = 10) exposed to 0 mg/L AgNO_3_ and the sub-MIC (22.5 mg/L). Yeast exposed to 0 mg/L AgNO_3_ displayed typical growth over the 72 h [32]. Yeast exposed to the sub-MIC displayed significantly reduced growth (*p* < 0.05) compared to the controls. These results coincide with other data that show that excessive metal exposure decreases cell growth, resulting in growth curve abnormalities [33,34,35]. 

NS commercial yeast had no significant growth after 11 h of exposure, and NS naïve yeast had no significant growth after 4 h of exposure. However, based on spectrophotometer results, NS commercial yeast had significantly more growth than NS naïve yeast after exposure to the sub-MIC. 

### 3.3. Scanning Eletron Microscopy (SEM)

**Figure 4:**
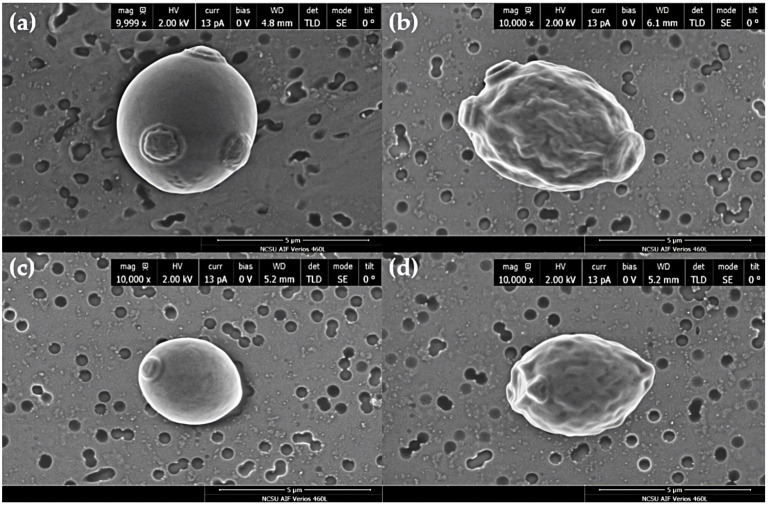
SEM micrographs of NS commercial and NS naïve yeast at 10,000× magnification. (**a**) and (**c**) are NS commercial and NS naïve yeast exposed to 0 mg/L AgNO_3_, respectively. (**b**,**d**) are NS commercial and NS naïve yeast exposed to 22.5 mg/L AgNO_3_, respectively.

**Figure 5:**
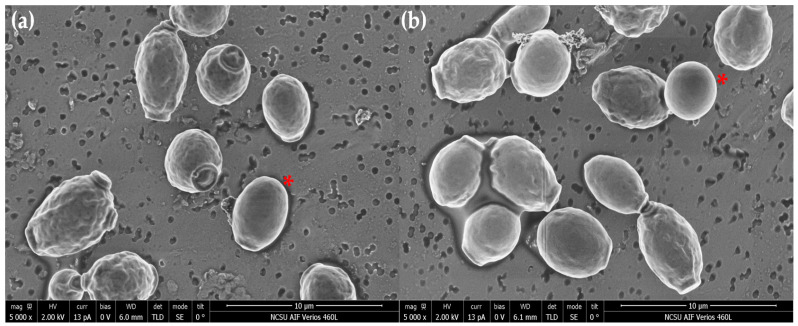
SEM micrographs of NS commercial (**a**) and NS naïve (**b**) yeast exposed to the MIC. The red asterisks indicate cells that do not have a drastic physical change and resemble controls.

SEM studies indicated a distinction in size between commercial and naïve yeast (Figure 4a–d, respectively), where commercial yeast was larger in size. Results also indicated that both yeast strains exposed to 0 mg/L AgNO_3_ (Figure 4a,c) had a smooth cell surface, typical morphology, for healthy *S. cerevisiae* cells [22,36]. However, exposure to the sub-MIC (Figure 5b,d) resulted in two distinct abnormal cell-surface morphologies:

1. A few cells had a smooth cell surface similar to unexposed yeast (Figure 5a,b).
2. A majority of cells had an abnormal physical appearance; cell surfaces of both yeast strains had drastic indentations or invaginations (Figure 5a,b).

### 3.4. Transmission Electron Microscopy (TEM)

**Figure 6:**
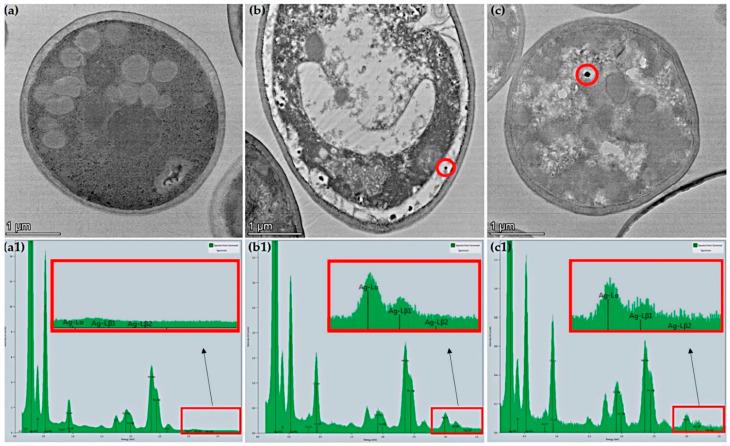
TEM micrographs of NS commercial yeast at 14,000× magnification. (**a**) depicts yeast exposed to 0 mg/L AgNO_3_. Both (**b**,**c**) depict yeast exposed to 22.5 mg/L AgNO_3_. The dark spots enclosed in a red circle in (**b**,**c**) are silver nanoparticles formed due to AgNO_3_ exposure. (**a1**,**b1**,**c1**) are the EDS spectrums that correspond to the image directly above them.

TEM analysis was also used to observe the physiological responses of yeast to silver exposure. The analysis produced results for NS commercial yeast; however, results for NS naïve yeast were inconclusive. Figure 6a shows micrographs for NS commercial yeast unexposed to AgNO_3_; typical morphology was observed, with no visible accumulation of any particles [37,38]. The corresponding energy-dispersive X-ray spectroscopy (EDS) spectrum showed no intracellular silver accumulation (Figure 6(a1)). Abnormal morphologies were observed in NS commercial yeast exposed to the sub-MIC, with two distinct morphologies:

1. Approximately 25% of cells appeared to have mild morphological differences (cytoplasmic reduction) compared to unexposed yeast, but overall similar appearance (Figure 6c).
2. Approximately 75% of cells displayed visible disruption of the cytoplasm, enlarged vacuole, and increased periplasmic space with irregular invaginations into the cytoplasm (Figure 6b).

NS commercial yeast exposed to the sub-MIC produced spherical/oblong silver nanoparticles that ranged from ~50 nm to ~90 nm in the widest axis end-to-end measurements (ImageJ 1.52 a). The presence of AgNPs was validated with corresponding EDS spectrums (Figure 6(b1,c1)). 

### 3.5. ICP-OES 

Due to the potential use of copper transport channels by silver ions and the potential induction of calcium signaling because of excessive metal exposure, ICP-OES was used to quantify the uptake of silver, copper, and calcium ions by *S. cerevisiae* after transient exposure to each (individually or in combination (copper and silver). ICP-OES was also used to determine if exposure to either treatment affected the ability of cells to take up the other (silver, copper, or calcium).

**Figure 7:**
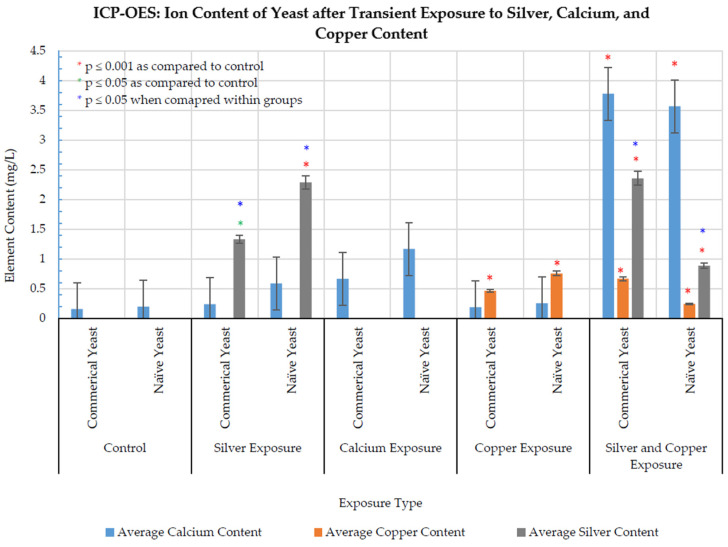
ICP-OES results of NS commercial (*n* = 5) and NS naïve (*n* = 5) yeast. Error bars represent percentage error (5%).

Results (Figure 7) indicated that transient silver exposure resulted in a significant increase in intracellular silver concentrations in both yeast strains, which indicated the ability of yeast to internalize silver ions via some uptake mechanism. Naïve yeast had a significantly higher silver concentration (~1.7-fold) than commercial yeast, which may have been due to the smaller size (observed in SEM micrographs) of the naïve yeast, which allowed for more cells to be present in a given volume, with a greater capacity for silver uptake per volume. Silver exposure did not significantly increase copper or calcium content in either strain. 

Transient copper exposure significantly increased intracellular copper concentrations in both yeast strains and did not significantly increase any other tested element. Transient calcium exposure did not significantly increase the intracellular concentrations of any tested elements. Exposure to equal concentrations of copper and silver had the most significant impact on elemental uptake, significantly increasing intracellular silver, copper, and calcium concentrations. 

## 4. Discussion

Fungal–silver interactions are rapidly increasing; however, fungal–silver homeostasis is only partly known. In the present study, two different, nonlaboratory *S. cerevisiae* strains were studied to determine the physiological impacts of transient silver exposure and if yeast ancestral backgrounds played a role in their response to AgNO_3_. 

MIC assays showed the ability of silver to inhibit all growth at 25 mg/L AgNO_3_ in both strains. SEM micrographs indicated that transient silver exposure physically impaired both yeast strains by causing an indented, cratered, shrunken appearance, similar to cells exposed to silver and other toxins [22,39]. These altered-morphology indicators coincide with the reduced cell-growth results observed in MIC and growth curve assays. Silver exposure is known to downregulate genes involved in ergosterol biosynthesis (*ERG3*, *ERG5*, *ERG6*, *ERG11*, *ERG25*, and *ERG2*), which may contribute to the observed altered appearance in SEM micrographs [16,22]. This result could also be a physical representation of osmotic stress or cellular dehydration [40,41]. Similar surface alterations have also been observed in *S. cerevisiae* exposed to other stressors, signifying that this morphological change may also be a general response to stress [39]. TEM imaging showed the accumulation of silver particles due to silver exposure and abnormal intracellular appearances. Similar results exist for *S. cerevisiae* exposed to 2 mM AgNO_3_ and the allergen 2S albumin [37,42]. Overall, TEM results indicated that NS commercial yeast had the capability to produce AgNPs after transient AgNO_3_ exposure, even at a level of silver exposure that is detrimental to cellular growth, demonstrating *S. cerevisiae* suitability for AgNPs mycosynthesis. ICP-OES demonstrated the capacity of yeast to uptake silver. Results indicated that silver exposure only affected intracellular silver concentrations, with no significant effect on calcium or copper uptake. Coexposure to copper and silver significantly increased calcium levels. This result agrees with data that show that exposure to toxic levels of cadmium and copper (independently) resulted in a sharp increase in calcium levels [43,44]. This indicates that calcium signaling may be a response to metal stress and may play an unknown role in metal resistance and tolerance [43,44].

## 5. Conclusions

All results indicated that silver exposure significantly altered physiology and cell morphology, with no bias in response based on *S. cerevisiae* strain ancestral backgrounds. *S. cerevisiae* was able to withstand exposure to varying concentrations of silver, internalize and make silver nanoparticles, which may make it a useful organism for silver nanoparticle production. However, this study was not focused on using *S. cerevisiae* for AgNP production. Instead, this first report is focused on the effect of AgNO_3_ on the growth physiology and morphology of *S. cerevisiae*. The current study is a prelude to further studies on the genomic and transcriptomic effects of AgNO_3_ in the yeast cells studied in the experiments reported here. A full-spectrum (physiology, genomics, and transcriptomics) study about metal interactions with *S. cerevisiae* will contribute immensely to understanding metal homeostasis and cellular function in model eukaryotic cells. Such essential and in-depth knowledge is critical for the engineering and manipulation of *S. cerevisiae* for better economic importance. 

## Figures and Tables

**Table 1:** Parameters for microwave-assisted acid digestion.

Digestion Parameters	Values
Power	800 W
Temperature	180 °C
Ramp Time	20 min
Hold Time	15 min

**Table 2:** Parameters for the elemental analysis by ICP.

ICP-OES Parameters	Values
RF Power	1500 Watts
Nebulizer Type	GemCone Low Flow
Nebulizer Gas Flow Rate	0.80 L/min
Plasma Gas Flow Rate	10 L/min
Auxillary Gas Flow Rate	0.25 L/min
Sample Flow Rate	1.60 mL/min

## Data Availability

The data presented in this study are available on request from the corresponding author. The data are not publicly available due to ongoing research on this subject.
